# Indigenous games and physical activity for preventing non-communicable diseases in African communities: a public health perspective

**DOI:** 10.3389/fspor.2025.1674875

**Published:** 2025-11-10

**Authors:** Musa Lewis Mathunjwa, Privilege Banqobile Ndlovu, Senzelwe Mazibuko, Gudani Goodman Mukoma, Dimitar Avramov

**Affiliations:** 1 Department of Human Movement Science, University of Zululand, KwaDlangezwa, South Africa; 2 Senzelwisihe Rehabilitation Hospital, Empangeni, South Africa; 3 Department of Biokinetics, Recreation, and Sport Science, Faculty of Health Sciences, University of Venda, Thohoyandou, South Africa; 4 Department of Sports, National Sports Academy “Vassil Levski”, Sofia, Bulgaria

**Keywords:** indigenous games, physical activity, non-communicable diseases, African communities, public health promotion

## Abstract

**Background:**

Rapid urbanisation and lifestyle shift in Africa have contributed to rising non-communicable diseases (NCDs) such as cardiovascular disease, diabetes, and certain cancers. While physical activity is a proven preventive measure, many interventions lack cultural resonance and affordability. Indigenous African games may offer a culturally relevant strategy to promote physical activity and reduce NCD risks. This paper discusses the possibility of applying such games across cultures and age categories and the pathway for knowledge transfer.

**Methods:**

A qualitative narrative review was conducted, synthesising peer-reviewed literature, public health data, and contextual case studies. The analysis focused on traditional games Ingqathu, Kgati, Diketo, and Morabaraba examining their physical, cognitive, and social benefits.

**Findings:**

Evidence shows that these games naturally incorporate movement, coordination, and community engagement, supporting cardiovascular health, enhancing metabolism, and reducing sedentary behaviour. Beyond physical benefits, they foster cultural identity, intergenerational interaction, and psychosocial well-being often overlooked in conventional exercise programmes. However, barriers such as erosion of traditional knowledge, lack of institutional support, and limited policy integration impede widespread implementation.

**Conclusion:**

Indigenous African games present a culturally grounded, cost-effective approach to increasing physical activity and mitigating NCD risk across diverse age groups. To maximise impact, revitalisation efforts should integrate these games into schools, community programmes, and public health policies to facilitate application and knowledge transfer. Future research should include intervention trials and scalability studies to confirm effectiveness and guide policy adoption.

## 1 Introduction

Cardiovascular diseases, diabetes, cancer, and chronic respiratory conditions are the most common types of non-communicable diseases (NCDs), and they are a leading cause of morbidity and mortality globally, particularly in low- and middle-income countries (1, 2). In the WHO African region, NCDs accounted for 37.1% of all deaths in 2019, a significant increase from 24.2% in 2000 (3). The burden of NCDs in sub-Saharan Africa grew by 67% between 1990 and 2017, and in 2019, approximately 1.6 million people aged 30–70 died prematurely from one of the major NCDs (4). This increase is also reflected in the Disability-Adjusted Life Years (DALYs), with the proportion of total DALYs attributable to NCDs rising from 18% to 30% during the same period. In South Africa specifically, NCDs accounted for 43% of total deaths in 2012, and the probability of dying from an NCD between the ages of 30 and 70 was 27% (5).

In African rural and peri-urban communities, this burden is increasing due to rapid urbanization, dietary shifts, and a reduction in physical activity (6, 7). Amidst populations in South Africa, where many rural populations lack access to basic healthcare and health education, the prevention and management of NCDs require low-cost and culturally appropriate strategies (8).

Increased physical inactivity is one of the main factors that are contributing to the rise of NCDs. Traditional lifestyles, which involved extensive walking, agriculture and communal labor, have been replaced by more sedentary habits, especially among younger generations (6). This shift has been further accelerated by the widespread availability of processed foods, technology-driven leisure activities, and migration to urban areas, which have led to higher rates of obesity and metabolic disorders (9, 10).

In response to this public health crisis, indigenous games are gaining recognition as effective tools for health promotion (2, 11). Games such as kgati, diketo, morabaraba, and ingculuza are deeply rooted in African culture and serve as a cultural heritage (12). They naturally promote motor behaviour (13) cardiovascular activity, coordination, and muscular endurance, while also fostering social cohesion and mental wellbeing (14).

Integrating indigenous games into community-based interventions and school curricula has the potential to reduce physical inactivity and related health risks. Such approaches foster sustainable, culturally sensitive health promotion initiatives, particularly in rural and peri-urban African communities (15). This paper aims to explore the potential of indigenous African games as culturally relevant and sustainable strategies for preventing NCDs. Specifically, it addresses: What are the physical, cognitive, and motor behaviour benefits, alongside social health benefits, of indigenous games? How can they promote physical activity to combat NCDs? What challenges, opportunities, and future research needs support their integration into public health programmes?

## 2 Methodology

The present paper employs a qualitative narrative review approach to synthesize existing literature available on indigenous games and their possible impact on physical activity and prevention of NCDs in African communities. This approach is justified by its utility in synthesizing diverse forms of evidence from case studies to public health data to provide a holistic, contextualised and interpretive view of an emerging public health topic (16). This methodology goes beyond a simple summary of outcomes to a thematic review of the literature, as previously established in similar public health literature (17).

### 2.1 Literature selection process and search strategy

The literature search was conducted across various academic databases such as PubMed, Scopus, Google Scholar, and African Journals Online (AJOL). Relevant peer-reviewed articles, reports, and books that were published within the past 15 years were identified using a combination of keywords and subject headings to make sure that the data is up-to-date. The search query was indigenous games OR traditional games OR African games then combined with a discussion on the interrelationship between these games and physical activities or health outcomes, and pertinent to the NCDs prevention or overall health in African populations. This review included peer-reviewed studies and credible reports on African indigenous games, especially those linking play to physical activity, health, and prevention of NCDs. Excluded were works outside the African context, unrelated to health, or non-academic sources, unless drawn from reputable official reports.

### 2.2 Thematic analysis approach

The literature gathered was subjected to a thematic analysis in order to discover, discuss and present trends or themes in the data. This was done through familiarization, preliminary coding of concepts of greatest interest to the health benefits of indigenous games and the sorting of this coding into higher themes of interest, including Physiological Benefits, Psychosocial Well-being and Community Engagement (12). This methodology guarantees the transparency and rigor of the review, which offers the structure of the discussion and conclusions of the manuscript.

## 3 Rationale for game selection

The choice of Kgati, Morabaraba, Diketo, and Ingqathu was deliberate, as each game represents a unique set of physical, cognitive, and social characteristics that together illustrate the broad potential of indigenous games as public health interventions (11).

Kgati (skipping rope): This is a dynamic, high intensity, and low-cost activity. The game involves one or more participants jumping over a rope that is swung in a circular motion, often to a rhythmic chant or song. It is a potent cardiovascular activity that builds endurance and agility (15). Its minimal equipment needs (a simple rope) and adaptability make it accessible across different age groups and ensures cultural relevance.

Morabaraba (The Twelve men's Morris/Strategic board game): Although less physically active, this two-player strategic board game is crucial for mental stimulation. Players move pieces (traditionally stones) to form “mils” (three-in-a-row) to capture the opponent's pieces. This rigorous cognitive engagement fosters strategy, problem-solving, and decision-making, which are essential in maintaining mental well-being and addressing stress (18).

Diketo (Stone Game): This is a skill-based game that enhances hand-eye coordination, fine motor skills, and sustained concentration. The basic objective involves tossing one stone into the air and simultaneously manipulating a set of other stones (typically five) on the ground before catching the tossed stone. This demonstrates how indigenous games simultaneously support physical dexterity and mental sharpness (18).

Ingqathu (Group Chase/Tag Game): This is an active, running-based group game often described as similar to tag or hide-and-seek. It highlights the social dimension of health by requiring continuous movement in a defined space, often with clearly defined roles for “chasers” and “runners”. By promoting teamwork, communication, and cohesion, it strengthens community bonds, which are vital in sustaining engagement in healthy activities and reducing isolation (18, 19).

Together, these four games demonstrate the holistic health value of indigenous play traditions.

To improve clarity and provide a systematic overview, the Table 1 summarizes the key characteristics of the four indigenous games discussed in this paper: Kgati, Morabaraba, Diketo, and Ingqathu. This comparison highlights their potential as culturally relevant and multifaceted tools for public health interventions.

**Table 1:** Comparative analysis of selected indigenous games.

Characteristic	Kgati	Morabaraba	Diketo	Ingqathu
Internal Logic: Roles	Rope swingers (2), Jumper(s) (1 or more), Chanter (often one of the swingers).	Two players (Opponent A & B), with alternating turns.	Tossing Player (Active player), Other players (Wait their turn).	Chasers (The “It” player/s), Runners/Hiders (The rest of the group).
Internal Logic: Sub-roles	Rhythmic timing, footwork, call-and-response chanting.	Strategic piece placement, ‘mill’ formation, defensive blocking.	Manipulating stones in specific patterns, catching the tossed stone.	Evasion, strategizing hiding spots, communication for team-tagging variants.
Internal Logic: Scoring/Winning	Successful completion of pre-agreed routines, number of jumps, or time without error (miss/trip).	The first player to capture a predetermined number of the opponent's pieces (typically 7).	Completion of a series of pre-defined stone-manipulation stages without error.	The Chaser tagging all Runners/Hiders, or the Runners successfully avoiding being tagged for a set duration.
Playing Style	High-intensity, cardiovascular, rhythmic, and aerobic.	Strategic, cognitive, and mentally challenging.	Skill-based, requires dexterity, hand-eye coordination, and concentration.	Active, running-based, collaborative, and social.
Individual vs. Group	Can be played individually or in groups.	Typically a one-on-one game.	Can be played alone or in a small group.	Exclusively a group game.
Age/Gender Relevance	All age groups, widely played by children and adults of all genders.	All age groups and genders.	Primarily played by children and young adults.	All age groups, widely popular among children.
Region	Widespread across Southern Africa and other regions.	Popular throughout Southern Africa, particularly among the Sotho and Tswana people.	Common in Southern Africa, with variations across communities.	Widespread across many African communities.
Required Equipment	A skipping rope. Can be made from simple materials like a piece of rope or old clothes.	A board with holes or markings and small stones.	A set of small stones (typically 5–10).	No equipment required.
Associated Costs	Very low to zero.	Very low to zero.	Very low to zero.	None.
Potential Risks	Minor risks such as trips or falls.	Minimal.	Minimal.	Minor risks such as falls while running.
Specific Health Effects	Improves cardiovascular endurance, agility, coordination, and muscular strength.	Enhances cognitive skills, strategic thinking, problem-solving, and mental well-being.	Improves hand-eye coordination, fine motor skills, and concentration.	Boosts cardiovascular health, social interaction, and teamwork.

## 4 The African communities burden of non-communicable diseases

African populations are progressively under pressure of NCDs with epidemiological dynamics indicating that cardiovascular disease, type 2 diabetes, cancer, and chronic respiratory diseases have burst in number (20, 21). Behavioural risk factors that fuel such trends are physical inactivity, poor dietary habits, tobacco smoking, and alcohol abuse (21, 22). Socioeconomic determinants and environmental factors are also significant in addition to lifestyle factors. NCDs are not diagnosed or well-managed as a result of poverty, poor access to healthcare and health literacy (22, 23). The high rate of urbanisation together with environmental shifts further enhances the lack of physical activities and the presence of unhealthy eating food options. Special vulnerability is given to the rural and peri-urban population because of poor infrastructure, the marginalisation of people and inequalities (23). To overcome the NCD burden in the African communities, the multisectoral approach should be employed including prevention strategies, early interventions, education, and community-based health strategies culturally appropriate (21, 23).

## 5 The rise of un-traditional lifestyles through physical inactivity

### 5.1 The world lives in the grip of inactivity crisis

Lack of physical activity has become one of the most serious global health problems which substantially contribute to the epidemic of NCDs. The World Health Organization (WHO) estimates over 1.4 billion insufficiently active adults across the world, with the worst case being in low- and middle-income countries (24). Such a crisis has an unequal impact on women and young people, especially in the urbanising societies where sedentary lifestyles are on the rise. Lack of exercise is associated with increased risks of heart diseases, obesity, diabetes type 2, some cancers and mental disorders (24, 25). These tendencies are becoming more noticeable in African countries, especially in rural as well as peri-urban areas, when the traditional types of physical labour are substituted with the passive ones with the use of transport, technology, and desk jobs (26).

### 5.2 Transition from indigenous to sedentary routines

These changes involved several transitions to sedentary lifestyles and other native forms of cultures. Traditionally residents of Africa led very active life, as they were farmers, they walked long distances, and fetched water, they rendered communal work. These routine activities were natural and continuous physical activity that was necessary in metabolic and cardiovascular health (27, 28). But with the urbanised, westernised lifestyles, these traditional ways have gone down. The emergence of processed food consumption, more time spent in front of screens, and fewer open recreation spaces has also led to the transition to sedentary lifestyles, in particular, to children and youth (29, 30). Loss of traditional games and dances, which is part of the erosion of the physical indigenous practice, is not only a lack of physical activities, but it is also a separation of the communities to their cultural roots. Establishing culture-based movement focused activities that are relevant to reinstating current health trends and maintaining cultural identity is necessary (31).

## 6 Indigenous games as a form of physical activity

Traditional games provide the most accessible and culturally appropriate type of physical activity, especially to peri-urban and rural Africans. Compared to formal sport which might involve organized facilities and gear, the native games are generally cheap, utilise materials available in the areas where they are played and are all inclusive regardless of age or gender (32, 33). There is cultural connection in these games, which maintains cultural knowledge base, social binding, and cross-generation communication (34). They are applicable to areas that are beyond play and play a role in the physical, cognitive and emotional growth. The indigenous games have a higher probability of acceptance and continuity in communities because they are local and founded on local traditions and values, thereby making them best to use in health promotion and physical activity interventions in low-resource communities (2, 21, 34).

## 7 Discussion

The analysis of indigenous games such as Kgati, Morabaraba, Diketo, and Ingqathu demonstrates that these activities are far more than recreational pastimes. They represent multidimensional tools that can support physical, mental, and social health. Unlike narrowly defined exercise programs, indigenous games integrate movement, cognition, and community engagement, thereby offering a culturally grounded and holistic approach to NCD prevention (35).

### 7.1 Physical health effects

Games such as Kgati and Ingqathu involve running, jumping, and bending, which elevate the heart rate, improve circulation, and enhance muscular endurance. These physiological outcomes align with global public health priorities aimed at reducing obesity, hypertension, and metabolic risk factors (36). Importantly, these games require little or no equipment, making them highly accessible to under-resourced communities where formal exercise facilities are scarce (37). By embedding health-enhancing activities within everyday cultural practices, they offer a low-cost and sustainable approach to tackling NCDs.

### 7.2 Psychosocial and cognitive effects

Beyond physical fitness, indigenous games also promote cognitive and psychosocial health. For instance, Morabaraba and Diketo stimulate memory, concentration, and problem-solving skills. Regular engagement in these mentally demanding activities may help delay cognitive decline and support healthy aging, particularly in societies facing a rising burden of age-related conditions (38). Furthermore, the enjoyment and social bonding experienced through gameplay reduce stress and foster resilience, which are protective against depression, anxiety, and cardiovascular disease (39, 40). Thus, indigenous games provide not only physiological benefits but also mental and emotional wellbeing.

### 7.3 Societal and cultural relevance

Perhaps the most unique contribution of indigenous games lies in their cultural embeddedness. These games are deeply rooted in African traditions, carrying symbolic and practical value that resonates with local communities. The act of playing itself is a form of cultural transmission, interpreting core values like cooperation (Ingqathu) and strategic patience (Morabaraba) not merely as skills but as life lessons embedded in play. Incorporating them into public health strategies validates indigenous knowledge, enhances community ownership, and reinforces cultural pride (2, 41). They also strengthen intergenerational ties, as elders transmit cultural interpretation, skills, and values to younger players (42). This collective approach reframes health not as an individual pursuit but as a shared social responsibility, aligning with broader calls for community-based solutions to public health challenges.

### 7.4 Case studies, policy, and challenges

Indigenous games represent a culturally grounded way of encouraging physical activity and reducing the burden of NCDs in African communities (2, 30, 43). Evidence from South Africa shows that games such as *kgati*, *diketo*, and *morabaraba* are not only enjoyable and accessible, but also provide low-cost opportunities to promote movement while strengthening cultural identity (11). When these activities are incorporated into schools or community health initiatives, participation tends to rise, particularly among women and children.

Although these case studies clearly indicate positive outcomes, they often lack the detailed intervention information needed to replicate or scale such programmes. For example, Dauenhauer et al.'s (44) work illustrates the success of after-school programmes where traditional games improved physical fitness, classroom behaviour, and social interaction. However, the exact intervention structure such as the number of weekly sessions or the duration of the programme was not specified (45). Similarly, Brand (19) describe township-based projects that enhanced motor skills, self-esteem, and cultural identity, while reports from Limpopo Province and KwaZulu-Natal highlight the use of *kgati* and *diketo* in school and weekend wellness activities. Yet, across these accounts, specific schedules remain unclear.

In contrast, Mathunjwa et al. (21) provide a well-defined 10-week Tae-bo programme that successfully improved fitness and reduced health risks. While informative, this example does not apply directly to indigenous games. This comparison underscores a critical gap in the literature: most indigenous game interventions document outcomes without clarifying programme length, frequency, or intensity. Addressing this limitation in future research would allow for stronger evidence, clearer guidelines for implementation, and the potential to expand these culturally relevant practices more effectively across diverse communities.

Policy frameworks in South Africa, including the Department of Sport, Arts and Culture's commitment to safeguarding indigenous knowledge, highlight the potential to institutionalize these games within physical education and community wellness programs. Such policies aim to complement formal health interventions and align with global calls for culturally sensitive health promotion strategies.

However, challenges remain. Limited funding, inadequate facilities, and a lack of structured integration into national health and education systems hinder widespread adoption. Additionally, there is a risk of undervaluing indigenous knowledge compared to Western-centric approaches. Sustained success requires strong policy backing, community ownership, and research that indicates the measurable health impacts of these games. Addressing these barriers will strengthen the role of indigenous games as effective tools in NCD prevention.

## 8 Conclusion

This narrative review aimed to explore the potential of African Indigenous Games as culturally relevant physical activity interventions to combat the rising tide of NCDs. The analysis revealed that games like Ingqathu and Morabaraba offer diverse, integrated physical and psychosocial benefits, providing new insights into an underutilized public health resource. Our findings bridge a critical gap by providing a theoretical framework for integrating Indigenous Games into policy. However, the study relies on preliminary evidence and case examples. Future research is essential: we urgently require Randomized Controlled Trials (RCTs) to establish the causal link between Indigenous Games and quantifiable health outcomes. Furthermore, the development of validated, specialized measurement tools is needed to accurately assess the unique physiological demands of these activities, paving the way for evidence-based implementation across African communities.
